# Applying Noncontact Sensing Technology in the Customized Product Design of Smart Clothes Based on Anthropometry

**DOI:** 10.3390/s21237978

**Published:** 2021-11-29

**Authors:** I-Jan Wang, Wei-Ting Chang, Wen-Hao Wu, Bor-Shyh Lin

**Affiliations:** 1 Department of Industrial Engineering and Enterprise Information, Tunghai University, Taichung 40704, Taiwan; ijwang@thu.edu.tw; 2 Department of Cardiology, Chi Mei Medical Center, Tainan 71004, Taiwan; weiting9901@stust.edu.tw; 3 Institute of Imaging and Biomedical Photonics, National Yang Ming Chiao Tung University, Tainan 71150, Taiwan; how840610@gmail.com; 4 Department of Medical Research, Chi Mei Medical Center, Tainan 71004, Taiwan

**Keywords:** electrocardiogram, smart clothes, dry electrode, customized product design, motion artifact, anthropometry

## Abstract

Electrocardiograms (ECGs) provide important information for diagnosing cardiovascular diseases. In clinical practice, the conventional Ag/AgCl electrode is generally used; however, it is not suitable for long-term ECG measurement because of the risk of allergic reactions on the skin and the dying issue of electrolytic gels. In previous studies, several dry electrodes have been proposed to address these issues. However, most dry electrodes, which are the mode of conductive materials, have to contact the skin well and are easily affected by motion artifacts in daily life. In the smart clothes developed in this study, a noncontact electrode was used to assess the biopotential across the clothes to prevent skin irritation and discomfort. Moreover, a three-dimensional parametric model based on anthropometric data was built, and the technique of customized product design was introduced into the smart clothes development process to reduce the influence of motion artifacts. The experimental results show that the proposed smart clothes can maintain a good ECG signal quality stably under motion from different activities.

## 1. Introduction

An electrocardiogram (ECG) is an important biosignal that reveals the electrical activity of the heart and can be used for diagnosing cardiovascular diseases, such as myocardial hypoxia, myocardial infarction, and arrhythmia. To access ECG signals, a conventional Ag/AgCl electrode is most commonly used in clinical research. In general, it is applied with electrolytic gels to improve the conductivity between the skin and the electrode [1]. However, using conventional Ag/AgCl electrodes with electrolytic gels for long-term ECG measurement can cause several problems, such as the dying issue of electrolytic gels or allergic reactions on the skin [2]. Therefore, using conventional Ag/AgCl electrodes with electrolytic gels is not convenient for long-term ECG measurements in daily life.

Recently, many dry electrodes that measure biopotentials without electrolytic gels have been developed. However, many dry electrodes still encounter the issue of higher electrode–skin interface impedance because of the poorer contact between the skin and the electrode. Various types of conductive material and structure have been applied in the development of dry electrodes [3,4,5,6,7,8,9,10,11], such as a carbon nanotube (CNT)/polydimethylsiloxane (PDMS) flexible electrode [12,13], 3D-printed electrode [14], and an ethylene propylene diene monomer electrode [15]. In 2012, Jung et al. proposed CNT/polydimethylsiloxane (PDMS) composite flexible dry electrodes [8]. A flexible PDMS substrate was covered with CNTs to improve its conductance. Because PDMS can provide better biocompatibility, this dry electrode contains a lower risk of skin irritation for long-term measurement. However, body hairs might affect the skin–electrode contact and interfere with the ECG signal quality.

In 2016, Pani et al. proposed a fully textile electrode [11]. The soaked conventional fabric, fabricated by placing it in a solution of poly3,4-ethylenedioxythiophene doped with poly(styrene sulfonate) (PEDOT:PSS), was cut into $30\times 30\;{\mathrm{mm}}^{2}$ and sewed on foam and polyester to form a fully textile electrode. This electrode could provide excellent performance for ECG measurement—as good as that of the conventional Ag/AgCl electrode. The flexibility of a fully textile electrode is comfortable, and the risk of skin irritation for the user is low. However, it is also easily affected by motion artifacts or fabric stretching, and even body hair might also affect the measuring performance. In 2017, Pei et al. proposed a skin-potential variation insensitive dry electrode [12]. This electrode is based on a microneedle array and made of silicon and coated with a titanium (Ti)/gold (Au) conductive layer by magnetron sputtering, with a size of $10\times 10\;{\mathrm{mm}}^{2}$. The root of the microneedles was insulated to reduce interference, and only the needle tips could access biopotential signals. Using the structure of microneedles to pass through the epidermis layer could effectively decrease the skin–electrode interface impedance and maintain higher stability and quality of biopotential measurement. However, these microneedles also have a high risk of breaking and may result in skin irritation or allergic reactions. The fabrication is relatively complex and expensive.

In contrast to the above dry electrodes, which have to contact the skin directly or invasively, capacitive electrodes have also been developed to access biopotential without direct contact with the skin directly [16,17,18]. However, these capacitive electrodes more easily suffer from the problem of motion artifacts, causing the triboelectric effect and variation in capacitance. In some studies, a type of capacitive electrode with a humidification system has been investigated, which provides a high relative humidity to remove the static charge rapidly and obtain a clear ECG signal [19,20]. However, it requires larger electrodes that sacrifice the spatial resolution and are difficult to place on the standard ECG locations, and they are inconvenient to use. In 2012, Spinelli et al. introduced a fast recovery circuit in a capacitive electrode to recover the ECG signal from saturation quickly [21]. In 2015, Serteyn et al. used an injection signal to record the capacitance change of the skin–electrode interface under ECG measurement, and they then utilized back-end processing with prerecorded data related to the capacitance change of the skin–electrode interface to reduce the influence of motion artifacts [22]. However, this method is unsuitable for real-time ECG measurements.

In this study, smart clothes using noncontact ECG electrodes and customized product designs were developed to access real-time ECG signals in daily life, even under motion. Moreover, in contrast to other smart clothes that usually do not contain both custom clothes and human-body-related numerical monitoring, a 3D parametric model based on anthropometric data was constructed. A customized product design technique was introduced into the smart clothes development process. To improve the fit of the smart clothes, a large amount of 3D mesh data of the human body were collected, a parametric torso model approximating the human body contour was established, and computer-aided design was integrated for smart clothing appearance design and tailoring. In the proposed smart clothes, noncontact electrodes can be easily placed on the chest and stably acquire the ECG signal by utilizing the mechanical design of the smart clothes. Finally, the performance of the ECG signal under different motion activities (sitting, standing, walking, and running) was also investigated. The experimental results show that the ECG signal quality obtained by the proposed smart clothes is as good as that of the conventional electrode with conductive gels, and it can maintain a stable signal quality during different activities in daily life.

## 2. Methods

### 2.1. Design and Implementation of Smart Clothes

**Figure 1:**
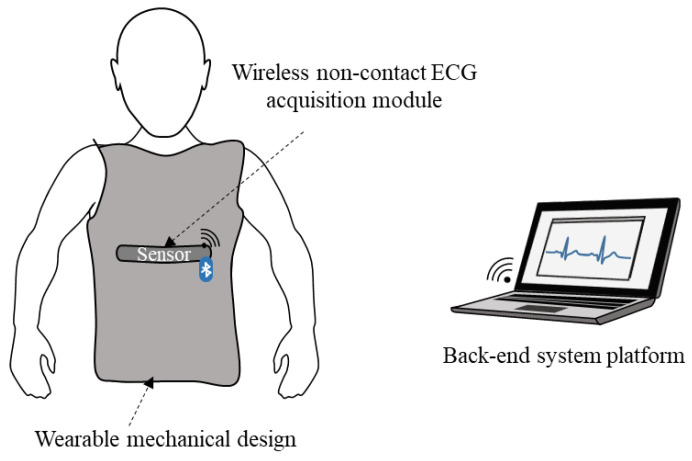
Basic scheme of proposed smart clothes system.

The system architecture of the proposed smart clothes is illustrated in Figure 1. It mainly consists of a wireless noncontact ECG acquisition module, wearable mechanical design, and back-end system platform. The wireless noncontact ECG acquisition module was designed to access the ECG signals across the clothes. It is made of flexible printed circuit boards (FPCBs), and its flexibility enables it to fit a round skin surface to access a good ECG signal quality. The wearable mechanical design has an embedded wireless noncontact ECG acquisition module and provides suitable pressure to ensure that the sensing module is close to the human body, even under motion in daily life. Finally, the acquired ECG signals are sent to the back-end system platform wirelessly via Bluetooth to display and store.

For the conventional Ag/AgCl electrode, conductive gels are usually applied in the gap between the epidermis and the electrode to reduce the equivalent impedance formed from the skin–electrode interface to acquire a higher-quality ECG signal. Therefore, in the equivalent electrical model for the electrode–skin interface of an Ag/AgCl electrode with conductive gels, the impedance of the conductive gel layer can be represented as a resistance. Because the epidermis also has the capacitance property [23], its model can be considered a parallel circuit of a capacitor and a resistance. Subcutaneous layers can be represented as resistance [24]. In contrast to conventional electrodes, the noncontact electrode can access biopotential without conductive gels, even across the clothes. In its equivalent circuit model, the clothing is an isolation layer, and the electrode and skin can be viewed as two parallel plates. Therefore, the skin–electrode interface can form an equivalent capacitor. The basic concept of the noncontact electrode is to acquire biopotential through capacitance formed from the skin–electrode interface, and using conductive gels and direct contact with the skin becomes unnecessary.

**Figure 2:**
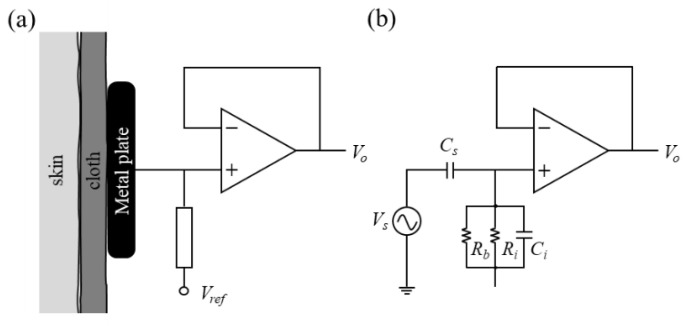
(**a**) Basic structure and (**b**) equivalent electrical circuit of noncontact electrode.

Figure 2a illustrates the basic structure of a noncontact electrode, which is mainly composed of an active circuit and a metal plate. Because the interface of the metal plate and skin can be viewed as a very small capacitance, an operational amplifier is designed as a unit buffer in the active circuit to provide an ultrahigh input impedance and avoid signal attenuation. The equivalent electrical circuit of the proposed noncontact electrode is shown in Figure 2b. Here, the source of the biopotential is presented as V_s_. C_s_ denotes the capacitance formed from the electrode–skin interface. R_b_ denotes the equivalent impedance of the bias pathway. The input resistance and input capacitance of the operational amplifier are represented by R_i_ and C_i_, respectively. The transfer function of the noncontact electrode can then be expressed as

(1) $$\frac{V_{o}\left(s\right)}{V_{s}\left(s\right)}=\frac{{\mathrm{sC}}_{s}}{s\left(C_{s}+C_{i}\right)+\frac{1}{R_{i}}+\frac{1}{R_{b}}}=\frac{{\mathrm{sC}}_{s}R_{i}R_{b}}{s\left(C_{s}+C_{i}\right)R_{i}R_{b}+R_{i}+R_{b}}\;$$

According to Equation (1), a better signal quality of biopotential measurement is obtained when the values of R_i_, R_b_, and C_s_ are increased or the value of C_i_ is decreased. The values of R_i_, R_b_, and C_i_ can be determined by selecting a suitable characteristic of the operational amplifier. An increase in C_s_ can be achieved by reducing the distance between the human body and the metal plate, or by expanding the area of the metal plate.

**Figure 3:**
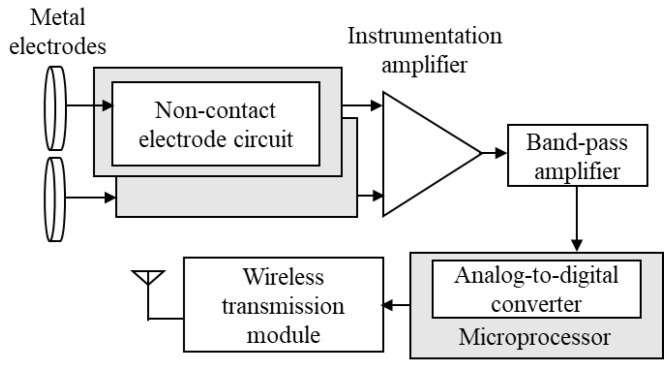
Block diagram of wireless noncontact ECG acquisition module.

Figure 3 is a block diagram of the proposed wireless noncontact ECG acquisition module. It contains several parts, such as metal electrodes, a noncontact electrode circuit, an instrumentation amplifier, bandpass amplifier, microprocessor, and wireless transmission module. First, the biopotential is accessed from the human body across the clothes using the noncontact electrode circuit with metal electrodes. Next, the acquired biopotential is amplified and filtered by an instrumentation amplifier (gain = 10) and a bandpass amplifier (gain = 50, frequency band = 0.1–100 Hz) to reserve the biopotential of interest and remove higher-frequency noise. In the microprocessor, a 12-bit analog-to-digital converter converts the preprocessed biosignal into digital biopotential data with a sampling rate of 500 Hz. Finally, the wireless transmission module transmits these digitized biopotential data to the back-end system platform.

### 2.2. Mechanical Design of Smart Clothes

In recent years, the technique of a 3D parametric model with a human body scanner has rapidly developed in clothing design to alleviate the issue of measurement error resulting from individual errors. In this study, a 3D parametric model based on anthropometric data collected by the 3D human body scanner (TG3D Scanatic 360 Body Scanner, TG3D Studio Inc., Taipei, Taiwan) was built, and the customized product design was introduced into the smart clothes development process. In contrast to conventional 3D scanners, the structure sensor of the 3D human body scanner used is compatible with tablets allowing fast and convenient data collection of anthropometric body dimensions for clothing design.

**Figure 4:**
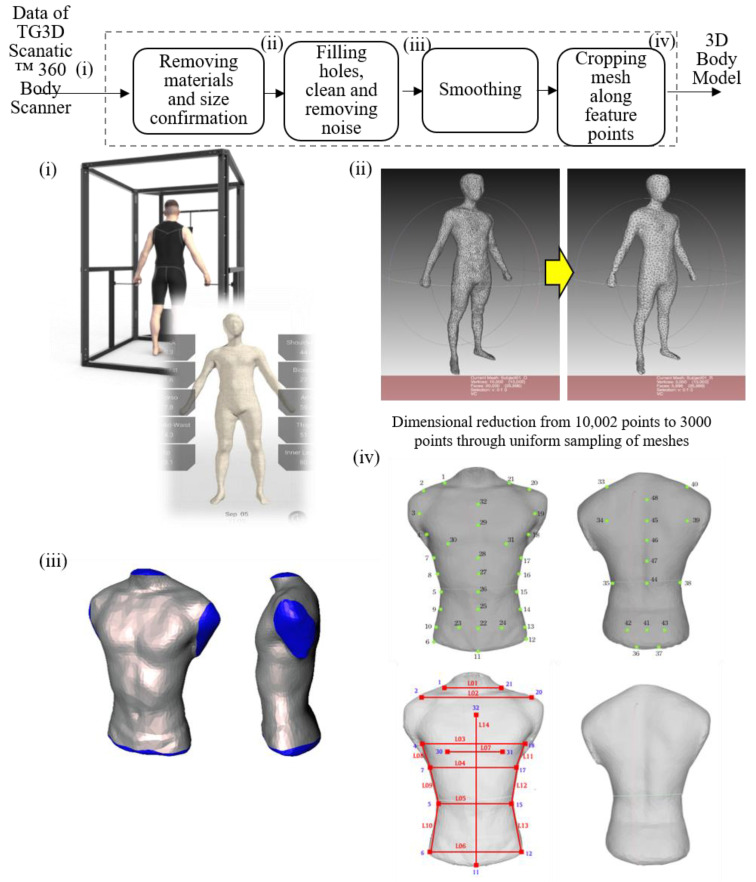
Illustration for postprocessing procedure of 3D human body geometry.

Although the scanner used has the advantages of a fast data capturing rate through noncontact measurement, the captured point groups occasionally exhibited poor quality, with several issues of overlapping, porosity, and noise. Therefore, data postprocessing is necessary to ensure consistency in the human body dimension database. Figure 4 illustrates the postprocessing procedure of the 3D human body geometry, which consists of several steps, including material removal, smoothing, cropping, patching, feature point selection, and cross-parameterization. Each of the raw datasets captured by the scanner contains tens of thousands of 3D coordinate points. However, there is no parametric model directly constructed from these raw sets because the model construction encounters excessive computational complexity. Therefore, the dimension of each dataset must be reduced to 3000 points through uniform sampling. Subsequently, the remaining meshes are processed through cross-parameterization. Because the meshes have to be enclosed, the holes on the boundaries are filled in this step. Finally, the codes of filling triangles are recorded to facilitate their removal after cross-parameterization to ensure a consistent number of meshes and connecting methods in each dataset.

**Figure 5:**
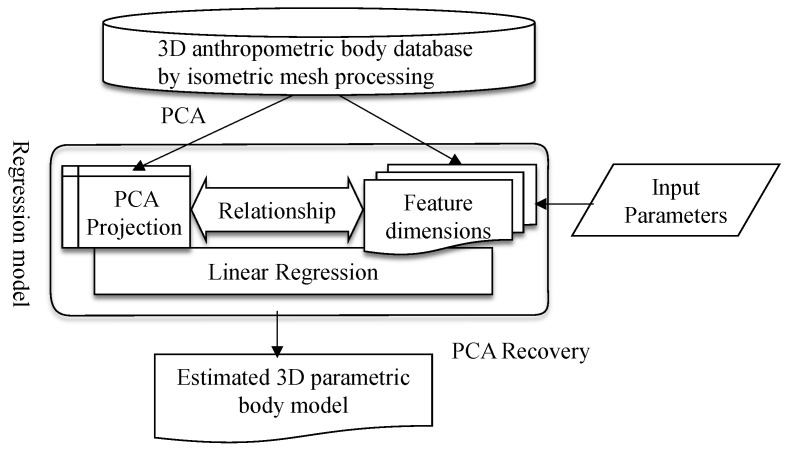
Procedure of parametric model construction.

Fourteen feature dimensions related to 3D human body dimensions [25] were selected for this study. The aforementioned 3D anthropometric database of the body is applied in the parametric model and the construction of correspondence between 3D coordinates and parameters through a regression model to generate editable parameters. The parametric model is intended to generate comprehensive 3D human body models through easily measured feature dimensions with a minimal number of parameters. Linear models are generally adequate for generating excellent results in the application of conventional parametric models with simple geometries. However, linear regression models may be inadequate for portraying detailed shape changes in complex parametric models of body parts, such as faces and bodies [26]. Therefore, a linear regression model is employed as the nucleus of the proposed parametric model, and the accuracies of the 3D models generated by these parametric models are compared, as shown in Figure 5.

### 2.3. Back-End System Platform

**Figure 6:**
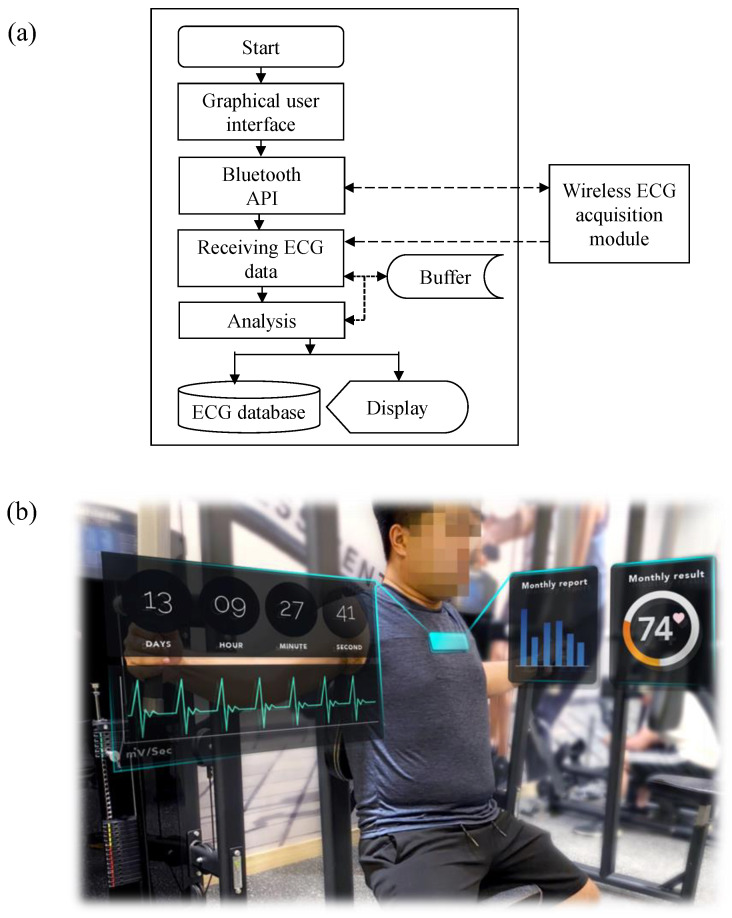
(**a**) Flowchart of proposed ECG monitoring program and (**b**) scenario diagram of the proposed smart clothes system.

A commercial laptop with the Windows 10 operating system was used as the platform of the back-end system. In the back-end system, the real-time ECG monitoring program is a Windows application developed using Microsoft Visual Studio. A flowchart of the proposed program is illustrated in Figure 6a. In the beginning, a graphical user interface enables the user to operate this system. When the user clicks the start button, the program calls the Bluetooth application programming interface to search for the wireless ECG acquisition module and connects it to this module. While connecting to the target device, the acquired ECG data are received by the back-end system to be analyzed and displayed in real time on the screen. In the back-end system analysis procedure, a 5-s moving window with a 3-s overlap is used to segment the raw ECG signal. Next, the locations and amplitudes of all *R*-waves in this segment are extracted. After all *R*-waves have been detected, the *R*–*R* interval, related to the heart rhythm, can be estimated. After the ECG analysis procedure, the raw ECG data, heart rhythm, and activity time are recorded. The platform of the back-end system can provide not only the current ECG analysis but also weekly or monthly reports of activities and exercises summarized from the ECG database. A scenario diagram of the proposed smart clothing system is shown in Figure 6b.

## 3. Results

### 3.1. Electrical Specification and Signal Quality of Flexible Noncontact Electrode

**Figure 7:**
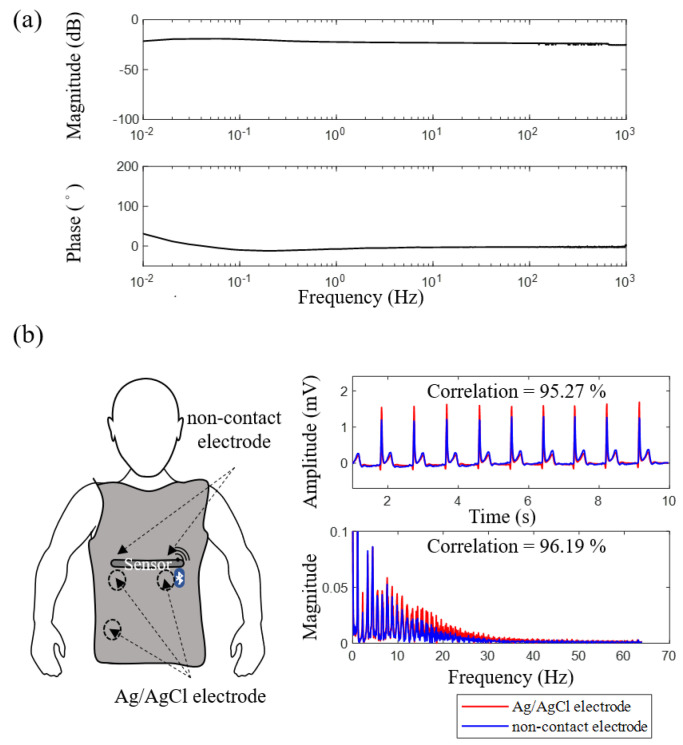
(**a**) Magnitude and phase responses of flexible noncontact electrode and (**b**) comparisons of ECG signals acquired by different electrodes in the time and frequency domains.

In this section, the electrical characteristics of the proposed noncontact electrode are discussed. In this experiment, metal plates covered with insulation tapes, connected to a function generator, were placed close to the proposed noncontact electrode. The function generator produced a 3-V peak-to-peak sinusoidal waveform with varying frequency (0.01–1000 Hz) to estimate the magnitude and phase responses. The experimental results are shown in Figure 7a. The magnitude response of the proposed noncontact electrode presents a stable response within the frequency band of 0.1–1000 Hz, and the phase response is almost linear within this frequency band. Therefore, the proposed flexible noncontact electrode is suitable for most biopotential measurements. The referred noise spectrum of the noncontact electrode was also investigated. In this experiment, the input of the noncontact electrode was connected to the ground. The referred noise spectrum of the output shows that the noise intensity of the entire frequency band was less than $10^{-3}V/\sqrt{\mathrm{Hz}}$.

To investigate the ECG signal quality obtained by the proposed noncontact electrode, the conventional Ag/AgCl electrode with conductive gels and the proposed noncontact electrode are compared in this section. In the experiment, the subject was instructed to wear the smart clothes to acquire lead-I ECG signals across the cotton clothes, and three Ag/AgCl electrodes were placed close to these noncontact electrodes simultaneously. The ECG signals obtained by different types of electrodes and their ECG spectra are illustrated in Figure 7b. The difference in ECG signal qualities acquired by different types of electrodes was estimated by the linear correlation coefficient using MATLAB. The correlation of the 3-min ECG signals obtained by different types of electrodes in the time domain was 95% and that in the frequency domain was 96%. For the frequency measurements of heart rate variability [27,28], the value of normalized low frequency power (nLF), normalized high frequency power (nHF) and LF/HF obtained by the proposed noncontact electrodes were 64.24, 35.76, and 1.80, respectively, and that of the conventional Ag/AgCl electrodes were 64.05, 35.95 and 1.78, respectively. The peak-to-peak amplitudes of the ECG signals obtained by the proposed noncontact electrodes and the conventional Ag/AgCl electrodes were 1.87 ± 0.43 (mV) and 1.89 ± 0.57 (mV), respectively. Next, for different types of electrodes, the comparison of the ECG quality collected from ten subjects was summarized in Table 1. According to the above experimental results, the ECG signals obtained by the proposed electrode and conventional Ag/AgCl electrode are highly similar.

### 3.2. Signal Quality of Smart Clothes under Different Motion Levels

**Figure 8:**
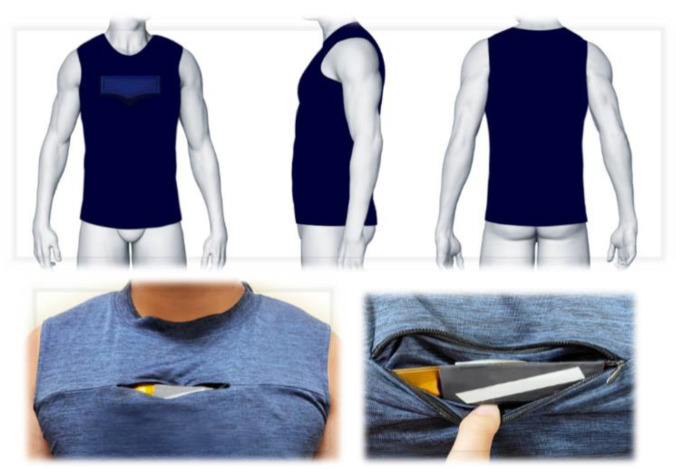
Simulation and photograph of smart clothes design based on parameterized 3D human body.

Smart clothes have the highest consumption rates among these products. The quality of the clothing design lies in the production of clothes that are close to the body. In this study, young Taiwanese men were used as subjects to establish a 3D human body database. The characteristic parameters of the human body were defined, and a linear regression model was adopted to establish the relationship between the parameters and a grid. Using this technique and the body characteristic parameters, geometric shapes similar to the physical bodies could be generated swiftly. Using these shapes, the users could customize their clothes by drawing curves onto the body model, retrieving the area within the range of the curves, and using meshing techniques to minimize the length of the boundary curve and the sum of boundary vertex angles to smooth out the body model. Figure 8 shows the simulation and photograph of the smart clothes design based on the parameterized 3D human body.

**Figure 9:**
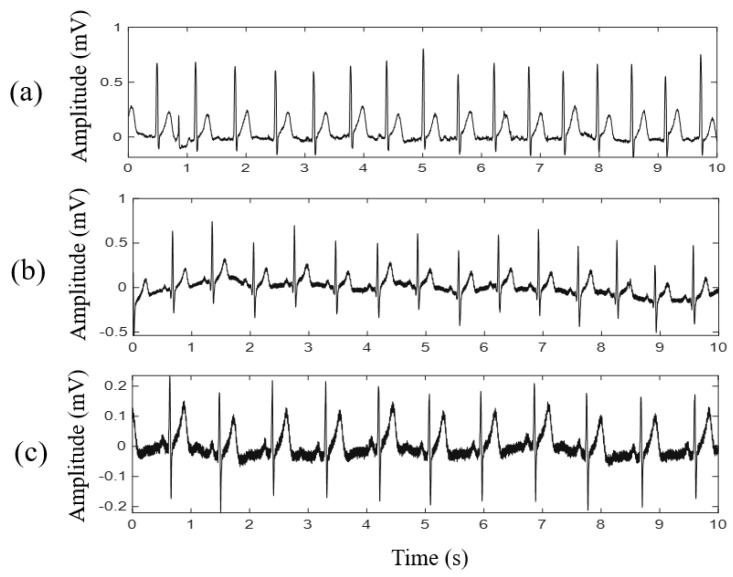
ECG signals acquired by different clothing designs, including smart clothes based on (**a**) parameterized 3D human body model and (**b**) average 3D human body model, as well as (**c**) commercial gym clothes with chest strap.

The efficiency of 3D human body modeling for ECG measurement using noncontact electrodes was investigated. The smart clothes based on the parameterized 3D human body model, the average 3D human body model, and commercial gym clothes with a chest strap were used for testing. The average 3D human body model was generated from the proposed 3D parametric model by averaging the coordinates of each feature dimension in all 3D human body models. According to Section 2.2, the 3D human body geometries of 20 subjects aged 18 to 22 were acquired using the 3D human body scanner. To reduce the possible bias when building the 3D parametric model, the body mass indices of the selected subjects were between 18.5 and 24.9, and the chest circumferences were 85.0–90.0 cm. This constraint avoided oversized or undersized clothing designs if a poor parametric model was generated because of the large differences between individual sizes. Moreover, a chest strap was used to fix the noncontact electrodes on the commercial gym clothes. Figure 9 shows the ECG signal qualities obtained from the different clothing designs. The ECG signal quality obtained by the smart clothes based on the parameterized 3D human body model was better than that of the average 3D human body model. The performance of the ECG measurement for the noncontact electrodes on commercial gym clothes with a chest strap was poor and unstable.

**Figure 10:**
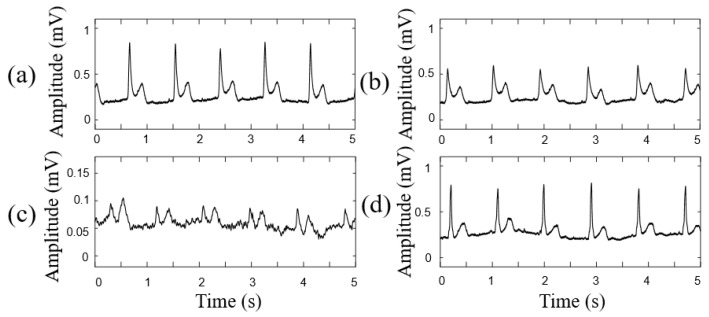
ECG signals acquired by smart clothing across the different thicknesses and materials: (**a**) 100% cotton, 0.35 mm, (**b**) 100% cotton, 0.7 mm, (**c**) 93% polyester, 0.31 mm, and (**d**) 100% cotton, 0.35 mm (sweating condition).

Next, the influence of the textile material and thickness of the smart clothes and sweating on the ECG measurement were investigated. The experimental results of the ECG measurement for different textile materials and thicknesses of the smart clothes are shown in Figure 10. The ECG signals obtained across different thicknesses (0.35 and 0.7 mm) of 100% cotton cloth were similar, but the amplitude of the ECG signal significantly declined with an increase in the textile thickness. However, the ECG signal obtained across the polyester cloth became very weak and unstable. Moreover, the influence of sweating in cotton cloth on the ECG measurements can be ignored.

**Figure 11:**
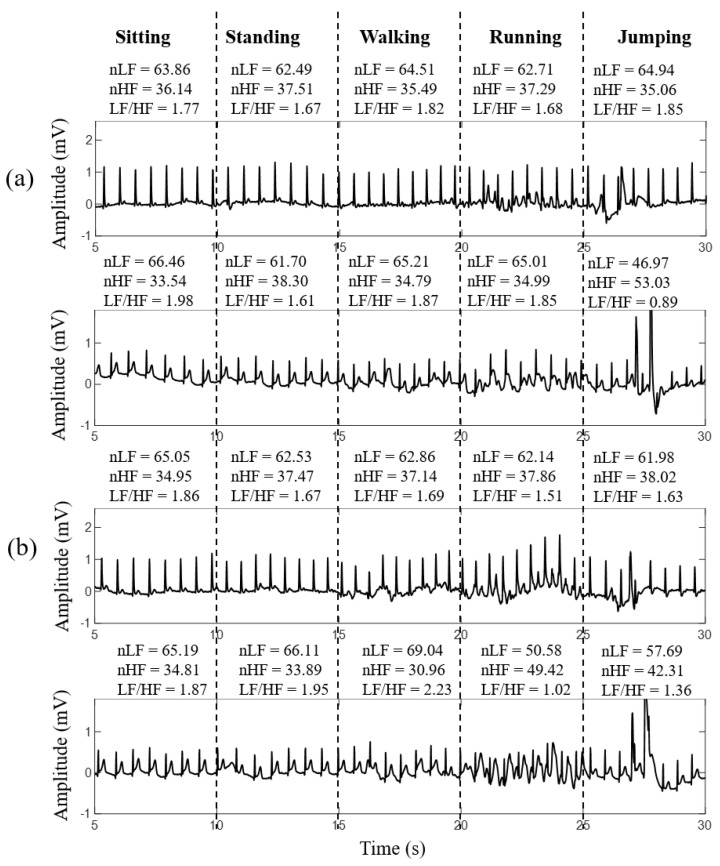
ECG signals of different participants acquired by (**a**) smart clothes and (**b**) conventional Ag/AgCl electrodes under different activities: sitting, standing, walking, running, and jumping.

Finally, the performances of the developed smart clothes and conventional Ag/AgCl electrodes under different daily activities and exercises, such as sitting, standing, walking, running, and jumping, were evaluated. The participants were instructed to perform different activities continuously every 5 s. The ECG signals acquired by the developed smart clothes and conventional Ag/AgCl electrodes are shown in Figure 11. Here, the ECG cable and Ag/AgCl electrodes were fixed by a chest strap to reduce the swinging of the cable during exercise. During sitting, standing, and walking, the quality of the ECG signal obtained by the developed smart clothes was stable, and the influence of motion artifacts was unapparent. However, during running and jumping, the baseline of the acquired ECG signal was slightly disturbed by motion artifacts, but the signal quality was still acceptable. The ECG signal quality obtained by conventional Ag/AgCl electrodes during walking remained acceptable, but it became poor during running and jumping.

## 4. Discussion

The experimental results in Figure 7a reveal that the magnitude response and phase response of the proposed noncontact electrode were stable and linear in the frequency band between 0.1 and 1000 Hz. The intensity of the referred noise was much less than $10^{-3}V/\sqrt{\mathrm{Hz}}$. According to the above specification, the proposed noncontact electrode is adequate for acquiring biopotentials, such as EMG, ECG, and EEG. The waveform and spectra of the ECG signal acquired by the proposed smart clothes were also compared with those acquired by the conventional Ag/AgCl electrode with conductive gels. The correlations between different types of electrodes in the time and frequency domains were both higher than 95%, as shown in Figure 7b. The experimental results validate that the proposed smart clothes can provide reliable ECG measurements.

Figure 9 shows the efficiency of the smart clothes based on the parameterized 3D human body model. The smart clothes based on the parameterized 3D human body model can fit the individual human body shape well and provide a suitable pressure on the noncontact electrodes. Therefore, the FPCB substrate of the proposed noncontact electrode can provide excellent flexibility to fit the human body shape and improve the ECG signal quality. The textile material and the thickness of the smart clothes affected the ECG signal quality. The cotton cloth was quite suitable for the noncontact electrodes because cotton has a higher permittivity than other common fabrics for clothing [29]. The signal quality becomes poor with an increase in the textile thickness, which can be explained by the capacitance formed from the electrode–skin interface becoming small when the textile thickness increases. The influence of sweating under intense exercise on ECG measurements can be ignored. Sweat may even improve the signal quality because the body sweat increases the permittivity of the coupling capacitor to decrease the capacitor impedance of the skin–electrode interface; therefore, the interference of the triboelectric effect caused by motion can be reduced.

The practicability and stability of the proposed smart clothes for ECG measurements under motion in daily life were also compared to those of conventional Ag/AgCl electrodes. The stability of the ECG signal quality obtained by the proposed smart clothes while walking was as good as that of sitting and standing. However, under some intense exercise (running and jumping), the motion artifact still caused a slight shift in the ECG baseline, but the ECG signal quality was still acceptable. The influence of intense exercise on the ECG measurement of the conventional Ag/AgCl electrodes was serious compared to the designed smart clothes because of the swinging of the ECG cable.

In previous studies, smart clothes were developed to monitor biopotential, and a comparison between other smart clothes and the proposed ones is listed in Table 2. In 2016, Trindade et al. developed smart clothing with a conductive textile electrode (electrode diameter of 16 mm) and an ECG data acquisition and transmission unit to monitor the ECG signal [30]. The T-shirt provided a good fit, and the conductive textile sensor had some advantages, including a lower risk of skin irritation for long-term ECG measurement and good washing durability. However, the conductive textile sensor had to contact the skin directly, and body hair, motion artifacts, and fabric stretching might affect the ECG signal quality. In 2015, Tada et al. proposed a smart shirt with a conductive ink electrode [31]. The sizes of the electrodes used were $20\times 30\;{\mathrm{mm}}^{2}$ or $20\times 20\;{\mathrm{mm}}^{2}$. The conductive ink provided excellent flexibility to maintain good contact conditions. The smart shirt with a conductive ink electrode also had a low risk of skin irritation and good washing durability, but stretching of the fabric could affect the signal quality. Moreover, sweat could cause short circuiting.

In 2017, Liu et al. proposed smart clothes using a CNT-PDMS polymer electrode, consisting of a vest sewed with a polymer electrode (electrode diameter of 30 mm) and a commercial ECG recorder [32]. The PDMS electrode had good biocompatibility to reduce the risk of skin irritation. However, this electrode still had to contact the skin directly. The measurement performance may be affected by a hairy site, and it could also be seriously affected by motion artifacts.

In this study, smart clothes were developed to monitor ECG signals using a noncontact sensing technique. In contrast to the above smart clothes, the proposed ones can effectively avoid skin irritation and reduce the influence of hair or sweat by accessing the ECG signal across the clothes. Moreover, the antimotion circuit of the ECG acquisition module and the mechanical design can effectively improve the measurement performance under motion in daily life. The proposed smart clothes design was developed using a noncontact sensing technique and the technique of a 3D parametric model with anthropometric data. Using the noncontact sensing technique, the proposed smart clothes can assess the long-term biopotential across the clothes to prevent skin irritation and discomfort. A 3D parametric model based on anthropometric data was built based on the body of the user to approximate the body geometry, and the technique of the customized product design was introduced into the smart clothes development process to reduce the influence of motion artifacts. Therefore, the designed smart clothes have great potential for monitoring ECG continuously in daily life. The wireless noncontact ECG acquisition module, wearable mechanical design of smart clothes, and back-end system platform in the proposed system were self-designed and self-assembled.

## 5. Conclusions

Novel smart clothes with a noncontact biopotential sensing technique were proposed to monitor ECG signals across clothes. The mechanical design of smart clothes can effectively reduce the occurrence of motion artifacts. The electrode specifications demonstrate that it is adequate to acquire most biopotentials. Moreover, the signal quality of the proposed noncontact dry electrode was as good as that of the conventional electrode with conductive gels (correlation > 95%). The experimental results show that the proposed smart clothes can maintain a stable and good signal quality under different activities in daily life. Compared to most of the current smart clothes that must contact the skin directly, the proposed smart clothes can avoid the problem of skin irritation for long-term ECG measurement and provide a better ECG signal quality under motion. Therefore, the proposed smart clothes have great potential for monitoring ECG continuously in daily life.

## Figures and Tables

**Table 1:** Comparison between ECG signal qualities obtained by different electrodes.

	Non-Contact Electrodes	Ag/AgCl Electrodes
nLF (nu)	64.60 ± 4.82	65.10 ± 4.70
nHF (nu)	35.40 ± 4.82	34.90 ± 4.70
LF/HF	1.87 ± 0.38	1.91 ± 0.40
Peak-to-peak amplitude	1.63 ± 0.51	1.71 ± 0.45
Signal correlation	94.96 ± 2.46	

**Table 2:** Specification comparison between other smart clothes and the proposed smart clothes.

	Smart Clothes with Textile Electrode [30]	Smart Clothes with Conductive Ink [31]	Smart Clothes with Polymer Electrode [32]	Proposed System
Area of electrodes (cm^2^)	2	4/6	7.07	14.96
Size of acquisition module	4 × 12 × 29.2 mm^3^	90 × 28 mm^2^	100 mm long	26 × 65 mm^2^
Frequency band (Hz)	0.05–100	-	-	0.1–100
Wireless transmission	Bluetooth	XBee	-	Bluetooth
Electrode material	Textile	Conductive ink	CNT-PDMS polymer	Copper
Non-contact electrode	No	No	No	Yes
Influence of motion	Larger	Larger	Larger	Smaller
Advantages	Lower risk of skin irritation, and good washing durability.	Lower risk of skin irritation, and good washing durability.	Good biocompatibility.	Non-contact measurement.
Limitations	Influence of hairs, motion artifact or fabric stretch.	Influence of fabric stretch and short circuit problem caused by sweat.	Influence of hairs and motion artifact.	Influence of cloth material and thickness.
